# Recommendations for future extensions to the HGNC gene fusion nomenclature

**DOI:** 10.1038/s41375-021-01458-0

**Published:** 2021-11-11

**Authors:** Alex H. Wagner, Yassmine Akkari, Marilyn Li, Angshumoy Roy, Karen Tsuchiya, Gordana Raca

**Affiliations:** 1 The Steve and Cindy Rasmussen Institute for Genomic Medicine, Nationwide Children’s Hospital, Columbus, OH USA; 2 Department of Pediatrics, The Ohio State University College of Medicine, Columbus, OH USA; 3 Legacy Laboratory Services, Legacy Health, Portland, OR USA; 4 Department of Pathology and Laboratory Medicine, Children’s Hospital of Philadelphia, Perelman School of Medicine at the University of Pennsylvania, Philadelphia, PA USA; 5 Center for Childhood Cancer Research, Children’s Hospital of Philadelphia, Philadelphia, PA USA; 6 Division of Pediatric Hematology and Oncology, Department of Pediatrics, Baylor College of Medicine, Houston, TX USA; 7 Department of Pathology & Immunology, Baylor College of Medicine, Houston, TX USA; 8 Department of Pathology, Texas Children’s Hospital, Houston, TX USA; 9 The Dan L. Duncan Comprehensive Cancer Center, Baylor College of Medicine, Houston, TX USA; 10 Department of Laboratory, Seattle Children’s Hospital, Seattle, WA USA; 11 Department of Laboratory Medicine and Pathology, University of Washington Medical Center, Seattle, WA USA; 12 Department of Pathology and Laboratory Medicine, Children’s Hospital Los Angeles, Los Angeles, CA USA; 13 Keck School of Medicine, University of Southern California, Los Angeles, CA USA

**Keywords:** Genetics, Genetics research

## To the Editor:

We are writing on behalf of a multi-consortia effort for the characterization of gene fusions as collaboratively defined by the Clinical Genome (ClinGen) Somatic Working Group, the Cancer Genomics Consortium (CGC), the Cytogenetics Committee of the College of American Pathologists (CAP) and the American College of Medical Genetics and Genomics (ACMG), and the Variant Interpretation for Cancer Consortium (VICC), a driver project of the Global Alliance for Genomics and Health (GA4GH). Our work (cancervariants.org/projects/fusions) is focused on disambiguating the many complex molecular events that constitute gene fusions and the molecular rearrangements that drive them.

Recently, Bruford et al. published the *HUGO Gene Nomenclature Committee (HGNC) recommendations for the designation of gene fusions* [1]. Recommendations by the HGNC, as a globally-recognized authority in the designation of human gene symbols, have provided a much-needed foundation for a unified nomenclature for human gene fusions, and we would like to congratulate the authors on this valuable publication. The primary recommendation from this manuscript is for a double-colon (“::”) delimiter for indicating fusions. This recommendation is conceptually aligned with other community recommendations, including the International System for Human Cytogenetic Nomenclature guidelines for genomic rearrangements [2], the HUGO Genome Variation Society nomenclature [3] for fusion transcripts, and guidelines for gene fusions in other organisms [4]. Our consortium thus supports the use of double-colon for fusion representation, and we will foster implementation of this HGNC recommendation within our participating organizations and the broader genetics community.

We now look to opportunities enabled by the HGNC gene fusion nomenclature recommendations to share our proposals for future expansion and refinement.

## The use of stable gene identifiers within fusion nomenclature

We look forward to an era where structured, computable metadata of genomic variation (including gene fusions) is routinely provided in clinical reports and published manuscripts, though this is unfortunately not the reality today. Utilizing only gene symbols to represent fusion genes (or native genes, for that matter) may lead to ambiguity or misinterpretation of nomenclature, as gene symbols continue to get updated. Therefore, there should exist a mechanism to unambiguously identify fusions represented by gene symbols only (e.g. *KMT2A*::*AFF1*) that is stable; one potential mechanism would be to use stable gene identifiers alongside the corresponding gene symbols, and in fact prior HGNC guidance recommends this very practice for the use of gene symbols in other contexts [5, 6].

While we acknowledge that the HGNC gene fusion guidelines explicitly “do not recommend that [HGNC gene] IDs be included in the fusion notation,” we see this as an opportunity for refinement in future versions of the guidelines. While there are undoubtedly scenarios where the mandatory and/or repeated use of HGNC IDs within the fusion nomenclature would be cumbersome, we envision scenarios where a standard representation for including identifiers alongside their associated symbols would be beneficial. For example, such conventions may help reduce errors by natural language processing tools seeking gene fusion events, especially if the fusion description is distant from identified gene symbols elsewhere in the manuscript or report.

## Differentiation of enhancer-driven and chimeric transcript fusions

One area for future expansion of the guidelines would be in the distinct representation of fusion partners describing a gene-associated regulatory element (e.g. an enhancer) vs. a chimeric RNA product. We commend the path being started in this direction by the HGNC recommendations, which recognize this distinction and promote a regulatory/enhancer convention for ordering indicated partners in regulatory fusions. Developing guidance for differentiating gene-associated regulatory elements from the genes they regulate may draw on previous nomenclatures that make this distinction [4].

## Differentiation of chromosomal rearrangements from gene fusions

Finally, we look towards greater clarity in future recommendations on the differentiation of gene fusions from the structural rearrangements that drive them, as these concepts are often conflated and used interchangeably in the literature and clinical reports. Further guidance and refinement in this area is still needed, including community consensus on if and when it is necessary to use hybrid notations (e.g. “*ABL1*::chr11.g:1850000” or “6q25::*ABL1*”) that mix chromosomal locations into a gene fusion nomenclature. Again we commend the steps taken by the HGNC guidelines to move in this direction by promoting the use of ISCN and HGVS nomenclatures for “formal reporting” of structural rearrangements.

## Conclusion

We appreciate the challenges of developing consensus recommendations, particularly in domains as complex and diverse as gene fusions and structural variation. The recommendations put forth by the HGNC are an advancement that, most importantly, is driving the community towards consistency in the representation of gene fusions. We look forward to future expansions of these recommendations which address the ongoing challenges in disambiguating this complex class of variation.
